# Methemoglobinemia and Delayed Encephalopathy After 5-Bromo-2-Nitropyridine Poisoning: A Rare Case Report

**DOI:** 10.3389/fpubh.2022.942003

**Published:** 2022-07-07

**Authors:** Longke Shi, Guangcai Yu, Liwen Zhao, Zixin Wen, Yaqian Li, Baotian Kan, Xiangdong Jian

**Affiliations:** ^1^School of Public Health, Cheeloo College of Medicine, Shandong University, Jinan, China; ^2^Department of Poisoning and Occupational Diseases, Emergency Medicine, Qilu Hospital of Shandong University, Cheeloo College of Medicine, Shandong University, Jinan, China; ^3^School of Nursing and Rehabilitation, Cheeloo College of Medicine, Shandong University, Jinan, China; ^4^Department of Geriatric Medicine, Qilu Hospital of Shandong University, Cheeloo College of Medicine, Shandong University, Jinan, China

**Keywords:** 5-bromo-2-nitropyridine, methemoglobinemia, hemolytic anemia, rhabdomyolysis, acute renal failure, toxic encephalopathy

## Abstract

5-bromo-2-nitropyridine, an intermediate in the synthesis of pharmaceutical and pesticide products, is toxic to the human body. However, 5-bromo-2-nitropyridine poisoning has not been previously reported. Here, we report the case of a 40-year-old man who suffered skin and respiratory tract exposure to leaked 5-Bromo-2-nitropyridine at work. After exposure, the patient rapidly developed dizziness, fatigue, nausea, vomiting, chest distress, diffuse cyanosis, and coma. Methemoglobinemia, hemolytic anemia, rhabdomyolysis, and acute renal failure were observed after admission. He improved markedly after treatment, but delayed encephalopathy was confirmed 82 days after the exposure. This case highlights that 5-bromo-2-nitropyridine can be absorbed through the skin and respiratory tract, resulting in methemoglobinemia and delayed encephalopathy.

## Introduction

5-bromo-2-nitropyridine (C_5_H_3_BrN_2_O_2_; CAS Number: 39856-50-3), also known as 2-nitro-5-bromopyridine and 3-bromo-6-nitropyridine, can cause irritation of the skin, eyes, and respiratory tract (1). It is a colorless or faint yellow solid at room temperature and is often used as an intermediate in medicine (2), pesticides, and the dye industry (3, 4). To our knowledge, the toxicologic features of 5-bromo-2-nitropyridine have not been previously reported. Here, we report the case of a patient with 5-bromo-2-nitropyridine poisoning through the skin and respiratory tract absorption who presented with methemoglobinemia, hemolytic anemia, rhabdomyolysis, acute renal failure, and delayed encephalopathy.

## Case Description

A 40-year-old man presented to the emergency department of a local hospital in a comatose state with dyspnea and cyanosis. He worked at a factory that manufactured 5-bromo-2-nitropyridine. Reportedly, white fog was generated in the workshop when the liquid form of 5-bromo-2-nitropyridine leaked from the faulty tank valve. Five minutes later, the patient was rescued from the workshop. At that time, white powder was observed on his bare face, neck, forearms and clothing; he felt a burning sensation and pain on his face, neck, and forearm. The patient wiped his bare skin with a wet towel 15 min after exposure. Thirty minutes after exposure, the patient developed dizziness, fatigue, nausea, vomiting, chest distress, tearing, and obvious cyanosis of the earlobes and lips. He was admitted to the hospital 2 h after exposure because of exacerbation of symptoms. During transit, the patient lost consciousness and had convulsions. Auscultation of the lungs and heart revealed no abnormalities; however, his oxyhemoglobin saturation was merely 70% (normal range 96–100%). Intravenous methylene blue (MB) 100 mg was immediately administered over 20 min, and endotracheal intubation with mechanical ventilation was performed. Arterial blood-gas analysis results were as follows: pH, 7.25; pO2, 503 mmHg; SaO2, 96.3%; lactate, 6.4 mmol/L; and methemoglobin (Met-Hb), 29.2%. The patient was transferred to the intensive care unit for further treatment.

An additional 80 mg dose of MB was administered, resulting in marked improvement in cyanosis and decreased Met-Hb to 3.6%. The main anomalies in the laboratory test results were as follows: alanine transaminase, 180 IU/L (normal value, 9–50 IU/L); aspartate aminotransferase, 548 IU/L (normal value, 15–40 IU/L); creatine kinase, >16,000 IU/L (normal value, 30–170 IU/L); creatine kinase-MB, 477 IU/L (normal value, 0–16 IU/L); lactic dehydrogenase, 4,264 IU/L (normal value, 313–618 IU/L); creatinine (Cr), 154 μmol/L (normal value, 57–97 μmol/L); and blood urea nitrogen (BUN), 10.3 mmol/L (normal value, 3.2–7.1 mmol/L) levels. Hemofiltration and intermittent hemoperfusion were performed. The patient dyspnea with increased airway secretion on day two after exposure, and bronchoscopy revealed mucosal edema of the main and segmental bronchus. Methylprednisolone (80 mg once a day) was administered. The tracheal cannula was removed on day three after exposure because the patient was conscious and had stable vital signs. However, he developed oliguria, and the Cr and BUN increased to 242.2 μmol/L and 13.3 mmol/L, respectively; therefore, continuous veno-venous hemofiltration was performed. On day five after exposure, he developed anuria, and Cr and BUN rose to 495 μmol/L and 28.9 mmol/L, respectively. On day seven after exposure, the patient was transferred to our hospital.

On admission, the patient was lucid, and his vital signs were as follows: temperature, 36.3°C; heart rate, 71 beats/min; respiratory rate, 20 beats/min; blood pressure, 140/82 mmHg; and oxyhemoglobin saturation, 99%. Findings from the physical examination were normal except for an anemic appearance and lower limb edema. Laboratory test results were as follows: hemoglobin, 65 g/L (normal range, 130–175 g/L); Cr, 810 μmol/L; and BUN, 42.8 mmol/L. Betamethasone (8 mg once a day), lansoprazole (30 mg twice a day), torasemide (20 mg twice a day), reduced glutathione (1.8 gram once a day), and alanyl-glutamine (10 g once a day) were administered daily. The patient's 24-h urine volume was 200 mL 24 h after admission; therefore, 6-h hemofiltration was performed. On day 10 after exposure, the 24-h urine volume was 280 mL, and the hemoglobin level had declined to 51 g/L; thus, two units of leukocyte-free erythrocytes were transfused. On day 13, the 24-h urine volume increased to 3,000 mL, hemoglobin level was 51 g/L, and 2 units of leukocyte-free erythrocytes were transfused again. On day 20, the patient was alert and oriented, and the hemoglobin level was restored to 72 g/L; hence, the patient was discharged from the hospital.

**Figure 1:**
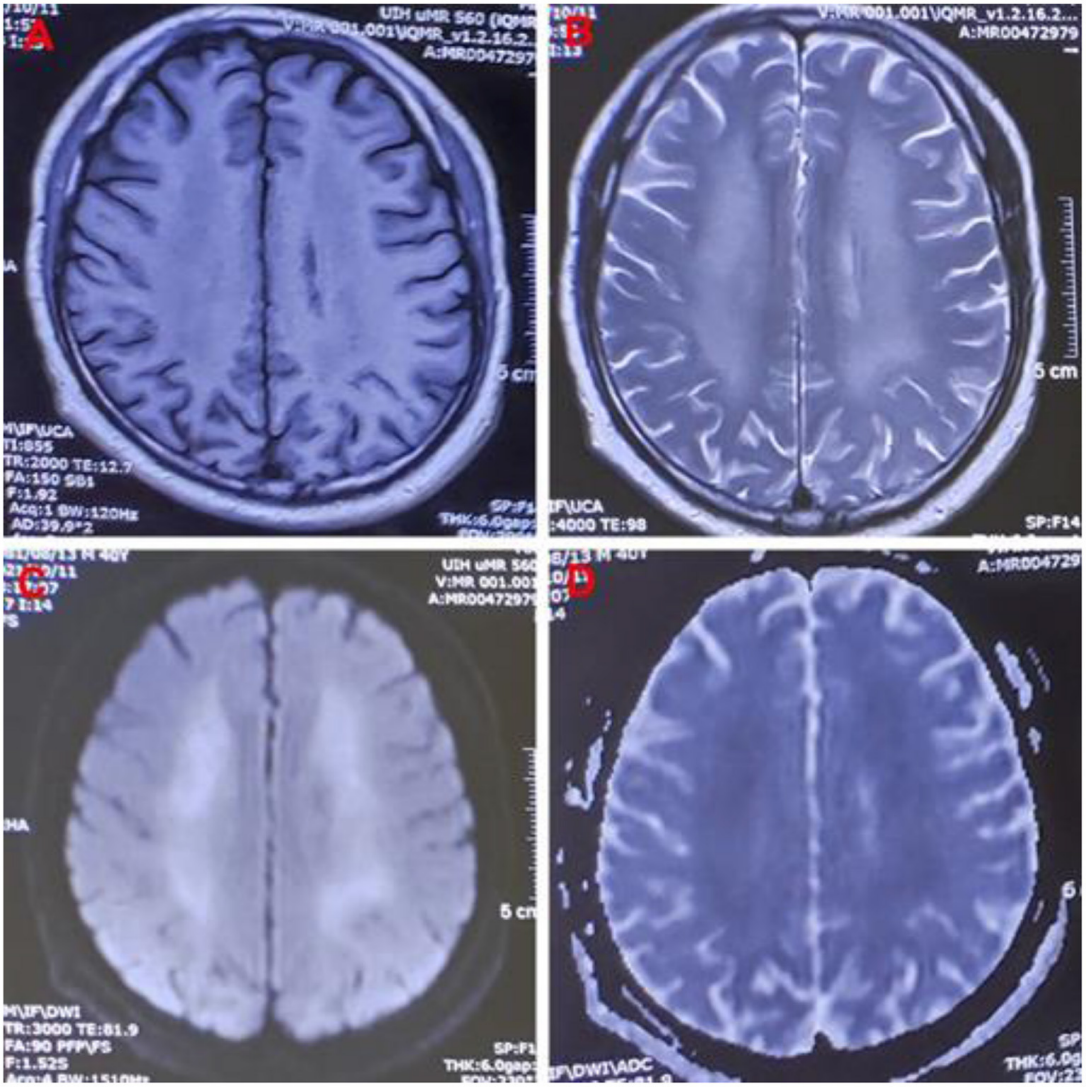
Images of a 40-year-old man exposed to 5-Bromo-2-nitropyridine. Magnetic resonance imaging 82 days after exposure showing abnormal signal intensity on **(A)** T1WI, **(B)** T2WI, **(C)** DWI, and **(D)** DWI/ADC in the bilateral cerebral hemispheres. T1WI, T1-weighted image; T2WI, T2-weighted image; DWI, diffusion-weighted imaging; DWI/ADC, diffusion-weighted imaging/apparent diffusion coefficient.

One month later, the patient complained of memory loss and slower motor responses. Electromyographic results were normal. Brain magnetic resonance imaging (MRI) showed symmetric abnormal signals [hypointensities on T1-weighted image (T1-WI) and hyperintensities on T2-weighted image (T2WI) and diffusion-weighted image (DWI)] in both the basal ganglia, corona radiata region, centrum semiovale, and thalamus; thus, a diagnosis of delayed encephalopathy was considered (Figure 1). Four months after exposure, central nervous system symptoms showed slight improvement, and brain signal abnormalities also improved. The patient's laboratory test results are shown in Table 1.

**Table 1:** Laboratory test results.

**Biochemical blood indicators**	**Local hospital**								**Our department**						
	**Normal values**	**8.20**	**8.21**	**8.21**	**8.21**	**8.22**	**8.23**	**8.2**	**Normal values**	**8.27**	**8.29**	**9.02**	**9.8**	**10.10**	**12.28**
		**10:18**	**1:26**	**9:19**	**21:06**	**9:32**	**12:34**	**5**		**18:37**	**8:51**				
WBC (×10^9^/L)	3.5–9.5	23.07	20.79	24.4	N	N	20.3	19.4	3.5–9.5	18.96	18.51	17.14	6.95	8.99	6.83
NEU (%)	40–75	86.2	85.0	87.9	N	N	88.8	84.9	40–75	83	81	79.8	94.4	72.1	66.5
RBC (×10^12^/L)	4.3–5.8	5.07	4.63	4.12	N	N	3.5	2.3	4.3–5.8	1.88	1.47	1.38	1.87	3.18	4.34
HGB (g/L)	130–175	159	140	123	N	N	107	79	130–175	65	51	51	72	102	132
PLT (×10^9^/L)	125–350	256	88	55	N	N	60	110	125–350	135	161	232	208	218	180
ALT (U/L)	9–50	180	367	387	360	290	252	148	21–72	91	49	48	145	12	16
AST (U/L)	15–40	548	789	589	308	116	105	70	17–59	51	22	18	48	14	16
DBIL (μmol/L)	0–5	N	N	7.9	0.9	5.4	5.6	13.3	0–5	16	18	7.4	5.0	2.1	3.4
IBIL (μmol/L)	0–19	N	N	18.8	18.4	27	42.6	42.2	0–19	35	18.8	3.6	2.9	3.8	5.4
CK (U/L)	30–170	>16,000	>16,000	20,288	13,079	732	1,600	3,332	55–170	758	366	1,225	34	49	57
CK-MB	0–16 (U/L)	477	335	266	287	N	29	N	0–4 (ng/ml)	4.2	3.6	3.0	4.5	0.7	0.6
LDH (U/L)	313–618	4,264	5,292	4,379	3,441	1,885	2,150	1,848	120–230	940	839	720	477	247	199
BUN (mmol/L)	3.2–7.1	10.3	11.3	12.8	13.9	13.3	15.6	28.9	3.2–7.1	42.8	49.1	58.3	48	4.4	5.8
Cr (μmol/L)	57–97	154	165.1	182.6	213.8	242.2	280.8	495	62–115	810	839	1,225	813	123	110

*ALT, alanine transaminase; AST, aspartate aminotransferase; BUN, blood urea nitrogen; CK, creatine kinase; CK-MB, creatine kinase-MB; Cr, creatinine; DBIL; direct bilirubin; HGB, hemoglobin; IBIL; indirect bilirubin; LDH, lactic dehydrogenase; NEU, neutrophils; PLT, platelets; RBC, red blood cells; WBC, white blood cells*.

This study was approved by the appropriate ethics committee of Qilu Hospital of Shandong University. Written informed consent was obtained from the patient's family.

## Discussion

The substance responsible for toxicity in the present case was 5-bromo-2-nitropyridine. After the patient was exposed to leaked 5-bromo-2-nitropyridine at work, he rapidly developed dizziness, fatigue, nausea, vomiting, chest distress, and coma. Meanwhile, the clinical and laboratory findings confirmed the diagnosis of methemoglobinemia. We speculate that 5-bromo-2-nitropyridine could oxidize hemoglobin to Met-Hb, causing cellular and tissue damage and oxidative hemolysis, as it has structural similarities with phenyl nitro compounds (5, 6). This could explain the rhabdomyolysis, hemolytic anemia, and acute renal failure observed in our patient.

Methemoglobinemia is caused by an increase in blood Met-Hb due to the conversion of ferrous ions to ferric ions in erythrocytes and results in tissue hypoxia and damage (7, 8). The most frequent causes of acquired methemoglobinemia are medications and other chemicals (9). Features of methemoglobinemia include diffuse cyanosis, chocolate-colored arterial blood (8, 10), unresponsive pulse oximetry saturation measurements, and elevated partial pressure of oxygen after supplemental oxygen administration (9). The results of pulse oximetry and arterial blood gas analysis were inconsistent with the aforementioned features (11). This may have been because pulse oximetry only measures the percentage of bound hemoglobin, which results in a false reading (12).

Hemolysis is the excessive destruction of erythrocytes, which can lead to anemia if not compensated for by adequate erythrocyte production. The causes of methemoglobinemia-related hemolysis include substance toxicity, glucose-6-phosphate dehydrogenase (G6PD) deficiency, and rapid or excessive overdose of MB (8, 13, 14). In our patient, methemoglobinemia caused histanoxia and muscle tissue damage; convulsions further exacerbated the muscle tissue damage, leading to rhabdomyolysis (7, 15). In this case, rhabdomyolysis and hemolysis occurred after 5-bromo-2-nitropyridine exposure, leading to a sharp increase in myohemoglobin and hemoglobin in the blood circulation and acute kidney injury. The patient showed marked improvement after treatment but developed slow movements and memory loss 2 months after exposure, and delayed encephalopathy was confirmed by MRI 82 days after exposure. The MRI showed symmetric hypointensities on T1WI and hyperintensities on T2WI and DWI in his basal ganglia, corona radiata region, centrum semiovale, and thalamus, which is similar to the findings in delayed encephalopathy after acute carbon monoxide poisoning (16). Some diseases can cause delayed encephalopathy (17), but in pyridine-compound poisoning, only a few cases involving toxic encephalopathy have been reported (18, 19). 5-bromo-2-nitropyridine-related delayed encephalopathy has not been previously reported, and the mechanism is unclear.

MB is the drug of choice for the treatment of methemoglobinemia; it reduces Met-Hb to hemoglobin through the NADPH Met-Hb reductase pathway, and the dose conventionally administered is 1–2 mg/kg (8, 20). For patients with G6PD deficiency, large doses of MB can worsen methemoglobinemia and lead to hemolysis; the effective lower dose of MB is 0.3–0.5 mg/kg (15). In our case, the initial total dose of MB may not be the main cause of hemolysis. In our previous report (8), the initial effective dose of MB for skin absorption-related methemoglobinemia was 40 mg (the two patients' weights were 55 and 85 kg). In this case, hemolysis still occurred even though our patient's Met-Hb reduced rapidly. Palliative care was administered to manage the hemolysis in addition to blood transfusions, if required, and prevent and treat complications. Once hemolysis and rhabdomyolysis have occurred, continuous blood purification treatment can effectively reduce hemoglobin and myohemoglobin in the blood circulation, thus protecting renal function (21).

In conclusion, 5-bromo-2-nitropyridine is toxic to the human body and can be absorbed through the skin and respiratory tract, which is likely to cause methemoglobinemia and delayed encephalopathy. In our patient, hemolytic anemia, rhabdomyolysis, and acute renal failure were mainly associated with 5-Bromo-2-nitropyridine-related methemoglobinemia.

## Data Availability Statement

The original contributions presented in the study are included in the article/supplementary material, further inquiries can be directed to the corresponding author/s.

## Ethics Statement

The studies involving human participants were reviewed and approved by Qilu Hospital of Shandong University. The patients/participants provided their written informed consent to participate in this study.

## Author Contributions

LS and XJ obtained research funding and investigated the description of the incident. GY and LS conceived the study and drafted the manuscript. LS, LZ, ZW, and GY supervised data collection. LS, GY, BK, and XJ take responsibility for the paper as a whole. All authors contributed substantially to its revision. All authors contributed to the article and approved the submitted version.

## Funding

This work was supported by the Qilu Hospital, Shandong University under Grant KYLL-2019-296.

## Conflict of Interest

The authors declare that the research was conducted in the absence of any commercial or financial relationships that could be construed as a potential conflict of interest.

## Publisher's Note

All claims expressed in this article are solely those of the authors and do not necessarily represent those of their affiliated organizations, or those of the publisher, the editors and the reviewers. Any product that may be evaluated in this article, or claim that may be made by its manufacturer, is not guaranteed or endorsed by the publisher.
